# Neuronal Polarity in the Embryonic Mammalian Cerebral Cortex

**DOI:** 10.3389/fncel.2017.00163

**Published:** 2017-06-16

**Authors:** Elif Kon, Alexia Cossard, Yves Jossin

**Affiliations:** Mammalian Development and Cell Biology Unit, Institute of Neuroscience, Université catholique de LouvainBrussels, Belgium

**Keywords:** neuron, polarity, neocortex, radial migration, multipolar migration, centrosome, reelin, ephrin

## Abstract

The cerebral cortex is composed of billions of neurons that can grossly be subdivided into two broad classes: inhibitory GABAergic interneurons and excitatory glutamatergic neurons. The majority of cortical neurons in mammals are the excitatory type and they are the main focus of this review article. Like many of the cells in multicellular organisms, fully differentiated neurons are both morphologically and functionally polarized. However, they go through several changes in polarity before reaching this final mature differentiated state. Neurons are derived from polarized neuronal progenitor/stem cells and their commitment to neuronal fate is decided by cellular and molecular asymmetry during their last division in the neurogenic zone. They migrate from their birthplace using so-called multipolar migration, during which they switch direction of movement several times, and repolarize for bipolar migration when the axon is specified. Therefore, neurons have to break their previous symmetry, change their morphology and adequately respond to polarizing signals during migration in order to reach the correct position in the cortex and start making connections. Finally, the dendritic tree is elaborated and the axon/dendrite morphological polarity is set. Here we will describe the function, establishment and maintenance of polarity during the different developmental steps starting from neural stem cell (NSC) division, neuronal migration and axon specification at embryonic developmental stages.

## Introduction: Development of The Cerebral Cortex

The process of brain development is quite amazing. In just 9 months, the human embryonic brain, which starts with a few hundred cells, undergoes a period of explosive growth that results in the generation of close to 100 billion neural cells. The right kinds of neurons have to be made at the adequate time and at the correct location. Each of these neurons has to make connections with an appropriate set of target cells in order to form the neural circuits that underlie the correct functioning of the brain. This precise establishment of neural connections occurs not just in the brain proper but also in the spinal cord and in the peripheral nervous system. However, we will limit this review article to an important part of the brain called the neocortex that plays key roles in learning and memory, sensory and motor functions and the control of our emotions. Cortical neurons can be excitatory, meaning that they trigger an electrical impulse to their targets, or inhibitory. Excitatory neurons are born in the neocortical ventricular zone (VZ) and migrate radially towards the pia in order to form the cortical plate (CP; Anderson et al., 2002; Gorski et al., 2002; Lui et al., 2011). Cortical inhibitory neurons are called interneurons and make local connections. Their main function is to gate or inhibit the initiation of an electrical impulse. Most of these cortical interneurons is born in the ganglionic eminences and migrates tangentially to reach the cortex (Hatten, 2002; Marin and Rubenstein, 2003; Hansen et al., 2013; Ma et al., 2013; Chu and Anderson, 2015). In the mammalian cortex, the majority of neurons are the excitatory type and we will mostly discuss this population of cells.

The cerebral cortex is the darker zone surrounding the white matter visible on coronal brain sections. This “gray matter” is composed of the cell bodies of different types of neurons that accumulate into six layers (Lui et al., 2011). Each layer comprises neurons that exhibit different morphologies, functions and properties. Excitatory neurons are either connected within the cortex in the same hemisphere or in a contralateral manner or connect to areas outside the cortex such as the spinal cord or the thalamus (Greig et al., 2013).

The formation of this layered structure relies on temporal ordering of stem cell self-renewal followed by well-regulated neurogenic divisions and finally the production of glia cells. Corticogenesis also depends on an extensive migration of immature neurons and their differentiation into fully functional neurons carrying an axon and multiple dendrites. These processes depend on correct polarization of the cells involved.

Polarity is a very important concept in cell biology. Cells are able to generate and maintain an asymmetrical and ordered distribution of structures along an axis resulting in asymmetric cell shape and/or cell functions. It involves a reorganization of cell-surface subdomains, the cell cytoskeleton, cellular organelles and proteins, and is usually triggered by external cues (Drubin and Nelson, 1996; Nelson, 2003). Polarity can be short-lived during dynamic processes or can be maintained for a protracted time such as the whole life of the cell. Virtually almost all cells in the body are polarized to some level and the establishment and maintenance of polarity is crucial during organogenesis.

**Figure 1:**
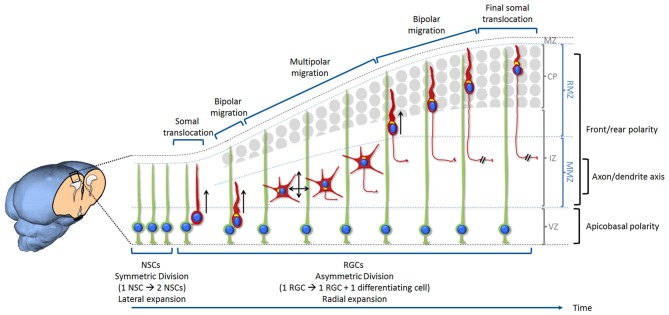
Overview of neurogenesis, migration and differentiation of glutamatergic neurons in the developing mammalian cerebral cortex. Neural stem cells (NSCs) are located at the ventricular zone (VZ). Initially, NSCs divide symmetrically in order to self-renew and increase their number. This results in an expansion of the pool of NSCs and a lateral expansion of the tissue. With the onset of neurogenesis NSCs progressively switch into radial glia cells (RGCs) and start dividing asymmetrically in order to self-renew and produce neurons. This starts the radial expansion of the tissue. During the very early stage of cortical development, the cerebral wall is thin and neurons migrate for a very short distance. After division, new neurons detach from the apical surface while they still keep a basal process inherited from the division of the elongated mother cell. The basal process shortens while nucleus and organelles translocate within the elongated cytoplasm. This particular migration is called somal translocation. Later on, with the accumulation of neurons above the VZ, the thickness of the cerebral wall increases and the somal translocation mode of migration becomes less frequent. It is replaced by a multi-phase mode of migration: first a very short bipolar migration when neurons exit the VZ followed by a multipolar migration in the multipolar morphology zone (MMZ), comprising the sub-VZ (sVZ; not shown here) and the lower part of the intermediate zone (IZ). This is followed by a bipolar migration called locomotion taking place in the radial morphology zone (RMZ) which is made up of the upper part of the IZ and the cortical plate (CP). When neurons reach the top of the RMZ, they seem to proceed through a glia-independent final somal translocation during which the leading process remains attached to the marginal zone (MZ) and shortens as the cell soma moves upward. Like for many other cell types, the centrosome and Golgi apparatus (represented in yellow in the figure) are facing the direction of migration. During the multipolar migration, the position of the centrosome and Golgi apparatus play an instructive role in specifying the future axon. Then the centrosome and Golgi apparatus move away from the growing axon in order to play their role in specifying the orientation of neuronal migration. The events described in this figure are influenced by three types of polarization. Neurons are produced by RGCs (under the influence of an apico-basal polarity) then migrate from their birthplace to their final destination (importance of a front-rear polarity) while the axon is specified during the multipolar phase (axon/dendrite polarity). Bipolar migrating cells (using somal translocation or locomotion) are moving towards the top of the CP (shown by a black arrow). Multipolar migrating neurons switch direction of migration several times tangentially and radially (both apically and basally) but the net movement is still directed towards the CP (shown by two crossing black arrows).

Intrinsic mechanisms of neuronal polarity have been investigated for a long time using cultures of hippocampal and cortical neurons and focusing on the axon/dendrite axis (Dotti et al., 1988; Craig and Banker, 1994; Tahirovic and Bradke, 2009). However polarity regulation *in vivo* is quite different, mostly because it occurs in a more complex tridimensional environment and is under the influence of a concerted action of intrinsic and extrinsic signals. In addition, other polarizing events besides the axon/dendrite axis are necessary before reaching the final mature differentiation state. Neurons are first produced by neuronal progenitor/stem cells (under the influence of an apico-basal polarity) then migrate from their birthplace to their final destination (importance of a front-rear polarity) while the axon is specified and finally dendrites are formed (axon/dendrite polarity; Figure 1). Cortical neurons undergoing these polarizing events travel through different regions of the tissue and therefore migrate through different extracellular environments and polarizing signals. In addition, a functional relationship exists between the molecular mechanisms underlying polarized migration and the final axon/dendrite polarity. Indeed, the trailing process of migrating cortical excitatory neurons is the future axon that elongates at the same time as the cell migrates. Similarly the leading process transforms into apical dendrites after completion of migration (Rakic, 1972; Schwartz et al., 1991; Hatanaka and Murakami, 2002; Hatanaka and Yamauchi, 2013). Because polarity is involved in all these events, defects in its establishment or maintenance have a tremendous effect on the correct functioning of the brain and result in a broad spectrum of disorders such as microcephaly, lissencephaly, mental retardation, schizophrenia, autism and epilepsy (Francis et al., 2006; Liu, 2011; Manzini and Walsh, 2011; Folsom and Fatemi, 2013; Ishii et al., 2016).

In spite of the knowledge accumulated over the years, it is still unclear how polarizing signals coordinate the different steps that pave the journey of a cell from its birth to its final settlement and differentiation into a fully functional neuron. In this review article we will discuss apicobasal polarity during division, front-rear polarity during migration and axon/dendrite polarity during differentiation of cortical excitatory neurons at embryonic developmental stage *in vivo*. We will mostly describe what we know of these events in rodents as most *in vivo* studies of mammalian cerebral cortex development have been done in mice.

## Polarity in Embryonic Neural Stem Cells and The Regulation of Neuronal Production

Excitatory neurons are produced by cortical neural stem cells (NSCs; Figure 1). Cortical NSCs contribute to most of the major cell types in the cortex: the different subtypes of excitatory neurons, astrocytes and oligodendrocytes (Campbell and Götz, 2002; Gorski et al., 2002; MuhChyi et al., 2013; Gallo and Deneen, 2014). NSCs line the ventricle in a region called VZ. Initially, they divide symmetrically in order to self-renew and amplify their number. This results in an expansion of the pool of NSCs and a lateral expansion of the tissue during embryonic day E9.5–E11.5 in mice (Takahashi et al., 1995).

Then, with the onset of neurogenesis, NSCs transform into another type of apical stem cells called radial glia cells (RGCs). RGCs continue to divide symmetrically, but asymmetric divisions are also observed. Asymmetric divisions create another RGC and a neuron (Miyata et al., 2004; Noctor et al., 2004). This starts the radial expansion of the tissue. With time, RGC division switches more often to the neurogenic asymmetric division. They also start producing intermediate progenitors or basal progenitors that migrate to the sub-VZ (sVZ) lying just above the VZ. Intermediate progenitors are restricted to one or two additional divisions, and all their progeny are neurons. This adds to the neuronal output from the RGCs (Haubensak et al., 2004; Sessa et al., 2008; Vasistha et al., 2015).

The sVZ that contains the intermediate progenitors is much larger in humans compared to mice and is divided into inner sVZ (ISVZ) and outer sVZ (OSVZ; LaMonica et al., 2012). An additional class of progenitor, called outer radial glia, has been described in the human OSVZ (Fietz et al., 2010). Outer radial glia are also present in ferret and mouse but at a much lower number. They still possess the long radial fiber attached to the pial surface but do not exhibit a process attached to the VZ. The increased diversity and number of progenitor cells in the human cortex account in part for its expansion and gyration (Hansen et al., 2010; Dehay et al., 2015).

Temporal regulation of the ratio of asymmetric to symmetric divisions has been intensively studied in cortical NSCs and RGCs and is critical for balancing appropriate neuronal production with progenitor/stem cells maintenance. Polarized distribution of cellular components or proteins has been investigated as the source of asymmetry in daughter cell fate specification.

### Apical Domain and Polarity Proteins

Cortical NSCs and RGCs exhibit an apicobasal polarity. Morphologically, they display a long pia-directed basal process that spans the entire cerebral wall and a short ventricle-directed apical process restricted to the VZ.

It was first suggested that asymmetric segregation of cellular components located at the apical domain between the two daughter cells during cytokinesis is important to regulate their fate. When only one of the daughter cell receives the apical domain, inheritance of this domain would lead to a self-renewing RGC while the other cell will begin migration and start differentiating into a neuron or intermediate progenitor (Kosodo et al., 2004; Marthiens and ffrench-Constant, 2009). In contrast, symmetrically dividing cortical NSCs would receive equal amounts of apical domain after division. However contradictory studies showed an equal distribution of the apical domain between the two daughter cells, even during asymmetric division, and demonstrated no correlation between cell fate of the two daughter cells and the ratio of their apical membrane size at cleavage (Konno et al., 2008; Noctor et al., 2008; Shitamukai et al., 2011). Because differentiating daughter cells inherit an apical domain, mechanisms important for the withdrawal of the apical process from the ventricular surface must come into play before they delaminate. One suggested mechanism is the downregulation of Adherens Junction (AJs) proteins (Rousso et al., 2012; Itoh et al., 2013). Here the transcriptional repression of cadherins is regulated by proneural genes expressed in differentiating daughter cells. In the chick and mouse spinal cord, abscission of the apical end-foot has been proposed as an alternative mechanism (Das and Storey, 2014). In this model, abscission is dependent on actin-myosin contraction and results in loss of apical cell polarity.

Molecularly, apicobasal polarity is established and maintained in part by cadherin-based AJs (Chenn et al., 1998; Harris and Tepass, 2010). The apical and basolateral membranes are separated by the AJs while occludin-based tight junctions are lost during the transition from apical NSCs to RGCs (Aaku-Saraste et al., 1996). AJs asymmetrically distribute proteins at the apical/basal dimension in cortical NSCs and RGCs while anchoring together the apical end-feet of adjacent cells (Margolis and Borg, 2005). The polarity protein mPar3 is enriched at the lateral domain in the ventricular end-feet of RGCs during interphase but shows asymmetric segregation as the cell cycle progresses. The daughter cell that accumulates mPar3 will become the RGC while the other one will differentiate (Bultje et al., 2009). mPar3 is a known apical polarity protein forming a complex with mPar6 and aPKC and able to interact with Cdc42 (Joberty et al., 2000; Lin et al., 2000). Studies on mPar6 (Costa et al., 2008) and Cdc42 (Cappello et al., 2006) suggest that these apical complex proteins are essential for self-renewal of neural progenitors in the developing mammalian cortex. However, in contrast to its role in invertebrates, deletion of aPKCλ in mice midway through neurogenesis did not clearly affect cell fate decisions, maybe because of some redundancy with aPKCζ (Imai et al., 2006). Pals1 belongs to another apical polarity complex and its conditional removal causes premature withdrawal from the cell cycle (Kim et al., 2010).

Basolateral complex proteins are also essential for the maintenance of cell polarity. Disruption of basolateral proteins and AJs proteins affects the stemness, the balance between symmetric and asymmetric division and the overall cell polarity in RGCs (Shen et al., 2002; Klezovitch et al., 2004; Petersen et al., 2004; Kadowaki et al., 2007; Rasin et al., 2007; Gil-Sanz et al., 2014). In mutant mice deleted for Llgl1, apical NSCs display loss of cell polarity and fail to divide asymmetrically, to exit the cell cycle and to differentiate (Klezovitch et al., 2004). These mice exhibit severe brain disorganization and hemorrhagic hydrocephalus leading to neonatal death. On the other hand, mice with conditionally deleted Llgl1 using Nestin-Cre recombinase survive and show symptoms of epilepsy. Their brains display severe periventricular heterotopias caused by disruption of apical junctional complexes (Jossin et al., 2017). Mechanistically, the basolateral protein LLGL1 directly binds to and promotes internalization of N-cadherin while LLGL1 phosphorylation by the apical protein aPKC prevents this interaction. In this model, the local concentration of N-cadherin in AJs located at the basolateral-apical boundary will increase, while kept low at the lateral membrane domain via LLGL1-mediated internalization (Jossin et al., 2017). Numb is enriched in the apical end-feet of RGCs (Rasin et al., 2007). Loss of function of Numb and Numbl using *in utero* electroporation disrupts cadherin-based AJs and polarity of RGCs (Rasin et al., 2007). Conditional deletion of both Numb and Numbl in the brain results in different effects on proliferation depending on the Cre used for the deletion. If the excision occurs around E8.5 (nestin-Cre) before the onset of neurogenesis, NSCs are depleted (Petersen et al., 2002). If the deletion occurs around E10.5 (D6-Cre) after the onset of neurogenesis, proliferative RGCs are strongly reduced in number (Petersen et al., 2004). However, ablation at around E9.5 using the Emx-Cre driver results in hyperproliferation of RGCs and a defect in differentiation (Li et al., 2003). This discrepancy could be explained by the timing and type of cells targeted (NSCs vs. RGCs) or the severity of the observed disorganization of the proliferative zone causing non-cell-autonomous effects.

Overall these results show that polarity proteins play crucial roles in cell fate decision and the control of proliferation.

### Basal Process

The basal end-foot of most NSCs and RGCs basal processes remains attached to the basal lamina throughout the cell cycle (Miyata et al., 2001; Noctor et al., 2001). Basal lamina, meninges, Cajal-Retzius cells and neurons constitute a niche that can signal to and influence NSCs and RGCs (Hartfuss et al., 2003; Seuntjens et al., 2009; Siegenthaler et al., 2009; Griveau et al., 2010). Moreover, the basal process may propagate signals from the end-foot to the cell body (Weissman et al., 2004; Rash et al., 2016). It is therefore not surprising that its inheritance has an influence on the maintenance of proliferative properties (Shitamukai et al., 2011). During the symmetric division of amplifying cortical NSCs, it can be split and equally divided among the two daughter cells (Kosodo et al., 2008), or it can be inherited by one cell while the other one re-extend its own basal process (Miyata et al., 2004). On the other hand, during the asymmetric division of RGCs, it is asymmetrically inherited by only one daughter cell that will self-renew whereas the other one differentiates into a neuron or an intermediate progenitor (Miyata et al., 2004; Shitamukai et al., 2011). Underscoring further its importance, it was shown that asymmetric distribution of Cyclin D2 mRNA at the basal end-foot ensures a RGC fate to the basally positioned daughter cell that inherits the basal process (Tsunekawa et al., 2012). More recently, Pilaz et al. (2016) showed that it is a site of active RNA movement and local translation.

All in all, it is possible that inheritance of both apical and basal processes is necessary to maintain a RGC phenotype.

### Centrosome and Primary Cilium

In cortical NSCs and RGCs, the centrosome functions as the basal body of the primary cilium located at the apical domain. The primary cilium is a small appendage of the plasma membrane poking into the cerebrospinal fluid and is central to sensing extracellular signals and transducing signaling cascades. Primary cilia are critical for normal brain development (Louvi and Grove, 2011). It seems that one of its functions is to determine the correct polarity of emerging RGCs. Indeed, disruption of the primary cilium activity that involves Arl13b results in a reversal of the apicobasal polarity of RGCs (Higginbotham et al., 2013). In these Arl13b mutant mice, cilia are present, but their function is impaired. RGC cell bodies are relocated from the VZ to near the pia surface, while glia end-feet-like structures are observed near the ventricle. The same group showed that conditionally deleting Arl13b later in development during the NSC to RGC transition period has less effect on the polarity and proliferation of RGCs, while deletion after the onset of neurogenesis does not affect RGC structure or division (Higginbotham et al., 2013). One of the signaling pathways conveyed by the primary cilium is Shh (Caspary et al., 2007; Rohatgi et al., 2007). Disruption of Gli3 function (a component of the Shh signaling cascade) after the onset of neurogenesis perturbs the regulation of RGCs proliferation without affecting apicobasal polarity (Wang et al., 2011; Wilson et al., 2012). All those results are suggesting that the primary cilium is important to determine polarity of NSCs and RGCs but is not necessary for its maintenance once it is set.

Molecular asymmetry is also observed at the level of the primary cilium and the associated centrosome during RGC division. A centrosome consists of a pair of centrioles (one mother and one daughter centriole) surrounded by an amorphous pericentriolar material. The two centrioles differ in structure and the older mother centriole supports ciliogenesis faster than the daughter centriole (Anderson and Stearns, 2009). During neurogenic asymmetric division, it was shown that the centrosome retaining the old mother centriole stays in the VZ and is preferentially inherited by RGCs, whereas the centrosome containing the new mother centriole is largely associated with differentiating cells (Wang et al., 2009). The primary cilium is dismantled during M phase and daughter cells have to re-establish it after mitosis completion. It was shown that the daughter cell receiving the older centriole re-form the primary cilium earlier than the other daughter and adopt a RGC fate (Paridaen et al., 2013). This asynchrony differentially exposes the daughter cells to primary cilium-transmitted signals and could explain the difference in fate.

In addition, the cell that inherits the younger centrosome forms a primary cilium located at the basolateral membrane instead of the apical membrane while still attached to the apical surface. The fate of this cell is to delaminate and become either a basal progenitor or a postmitotic neuron (Wilsch-Bräuninger et al., 2012). This asymmetrical inheritance of the primary cilium has consequences on the timing and quality of the signals it potentially receives such as proliferative cues present in the cerebrospinal fluid (Lehtinen et al., 2011).

In humans, diseases related to defects in primary cilia are associated with brain malformations and mental retardation (Rauch et al., 2008; Goetz and Anderson, 2010; Lee and Gleeson, 2011). Likewise, there are clear genetic links between centrosomal proteins and human brain diseases such as microcephaly (Woods et al., 2005; Kumar et al., 2009; Chavali et al., 2014). Notably, the multiple brain malformations observed in some microcephaly patients with specific mutations suggest that the centrosome and its associated proteins are involved in multiple aspects of brain development such as neurogenesis, polarity, migration or differentiation (Passemard et al., 2009; Nicholas et al., 2010; Yu et al., 2010).

## Polarity in Early Born Neurons

During the very early stage of cortical development, the cerebral wall is thin and neurons migrate for a very short distance. After division, new neurons detach from the apical surface while they still keep a basal process inherited from the division of the elongated mother cell. The basal process shortens while nucleus and organelles translocate within the elongated cytoplasm (Nadarajah et al., 2001; Gupta et al., 2002). This particular migration is called somal translocation (Figure 1). The polarity is already set after cell division and seems to be inherited from the apicobasal polarity of the RGCs. This process is very similar to what occurs during retinal ganglion cell migration (Zolessi et al., 2006; Randlett et al., 2011).

In parallel with the somal translocation, the axon grows from the apical side of the translocating neuron. It is possible that, similarly to what was shown for Drosophila sensory neurons, molecular remnants such as the clustering of AJ components corresponding to the last mitotic cleavage might be important for axon specification (Pollarolo et al., 2011).

## Polarity in Mid to Late Born Neurons

Later on, with the accumulation of neurons above the VZ, the thickness of the cerebral wall increases and the somal translocation mode of migration becomes less frequent. It is replaced by a multi-phase mode of migration: a very short bipolar migration when neurons exit the VZ followed by an extended multipolar migration occurring in the multipolar morphology zone (MMZ), comprising the sVZ and the lower part of the intermediate zone (IZ). This is followed by a bipolar migration called locomotion taking place in the radial morphology zone (RMZ) which is made up of the upper part of the IZ and the cortical plate (CP; Shoukimas and Hinds, 1978; Nadarajah et al., 2001; Noctor et al., 2004; Jossin, 2011). When neurons reach the top of the RMZ, they seem to proceed through a glia-independent final somal translocation during which the leading process remains attached to the marginal zone (MZ) and shortens as the cell soma moves upward (Nadarajah et al., 2001; Sekine et al., 2011; Figure 1).

During the short bipolar migration at the VZ, cells are polarized towards the CP. Similarly to what occurs for early born cortical neurons and during retinal ganglion cell migration (Zolessi et al., 2006; Randlett et al., 2011), they might inherit polarity from the mother neuroepithelial cell. This is visible by the asymmetric distribution of N-cadherin at one pole of the daughter neuron after division (Gärtner et al., 2012). However the axon is not specified at that stage. When they reach the MMZ, they become multipolar. “Multipolar neurons” means that they lose their bipolar morphology and extend more than two neurites. Those neurites extend and retract frequently and point to multiple directions. During this multipolar stage, they switch direction of migration several times tangentially and radially (both apically and basally) but the net movement is still directed towards the CP (Tabata and Nakajima, 2003; Bielas et al., 2004; Noctor et al., 2004; Figure 1). Nothing is known about the signals that triggers this bipolar to multipolar change of shape and polarity. One study however suggested the involvement of Connexin 43 (Liu et al., 2012). Another unanswered question is why they go through this multipolar stage. Multipolar migration might help neurons move across the IZ crowded with tangentially oriented axons but recent studies suggest a more functional role in tangentially dispersing cortical neurons (see below; Torii et al., 2009; Dimidschstein et al., 2013).

At the same time intermediate basal progenitors are produced and most of the late-born upper layer neurons come from these cells (Molyneaux et al., 2007; Sessa et al., 2008; Kowalczyk et al., 2009). Unlike RGCs, basal progenitors do not seem to possess an apicobasal polarity and therefore cannot provide a polarity cue to the multipolar neurons they give birth to.

### Extracellular Signals for the Directionality of Multipolar Migration

**Figure 2:**
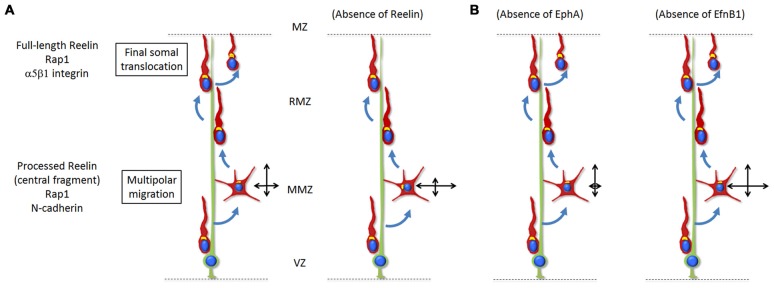
**(A)** Reelin signaling, via Rap1 and N-Cadherin, regulates the orientation of multipolar neurons towards the top of the CP. Only the processed protein and most importantly the active central processing fragment diffuse from the MZ into the deeper tissue such as the MMZ. The absence of Reelin signaling results in disoriented migration (represented by the two crossing black arrows) with a reduced movement towards the CP and increased lateral movement in the tangential directions. During the last stages of radial migration, Reelin signaling controls the final somal translocation via inside-out activation of α5β1-Integrin signaling. The absence of Reelin signaling results in a defective final somal translocation. **(B)** Ephrin-A/EphA forward signaling controls the lateral distribution of neurons by promoting a wider tangential migration during the multipolar stage. Absence of the signal results in a decreased lateral movement. On the other hand, ephrin-B1 reverse signaling has an opposite effect and restricts the tangential migration of mutipolar neurons at the MMZ. Absence of the signal results in a wider lateral movement.

While many regulatory pathways involved in the multipolar to bipolar migration transition have been investigated (briefly discussed in “Signals for Multipolar to Bipolar Transition” Section), only a few signaling pathways were proposed to regulate the directionality of multipolar neurons. Recently the extracellular protein Reelin has been shown to be involved in polarizing the movement of multipolar neurons towards the CP while they are migrating through the MMZ (Jossin, 2011; Jossin and Cooper, 2011; Figure 2). In the absence of Reelin (reeler mice), mid to late born neurons exhibit a broader and irregular distribution and fail to organize into layers (Boyle et al., 2011). In humans, mutations in the reelin pathway are associated with autosomal recessive lissencephaly and cerebellar hypoplasia, schizophrenia, autism and epilepsy (Hong et al., 2000; Chang et al., 2007; Folsom and Fatemi, 2013; Ishii et al., 2016). Reelin is secreted by Cajal-Retzius cells located in the MZ right above the CP (D’Arcangelo et al., 1995; Ogawa et al., 1995). Earlier studies showed that, while the full-length Reelin is mostly present near its secreting cells, only the processed protein and most importantly the active central processing fragment diffuse from the MZ into the deeper tissue such as the MMZ (Jossin et al., 2007). Interestingly, the MMZ is where the highest expression of receptors for Reelin is seen on migrating neurons (Uchida et al., 2009; Hirota et al., 2015). Later on, *in vivo* experiments and using time lapse videomicroscopy in organotypic and lattice cultures demonstrated that the inhibition of intracellular signaling induced by Reelin reduces the movement of multipolar neurons towards the CP and increases the tangential movements whereas the speed of migration is not affected (Jossin and Cooper, 2011). In parallel, fewer multipolar neurons point their Golgi apparatus (usually facing the direction of migration) towards the CP. Along this line, a function of Reelin in orienting the Golgi apparatus from the axon toward the future largest dendrite was shown *in vivo* and *in vitro* (Matsuki et al., 2010; Meseke et al., 2013).

The action of Reelin at the MMZ depends on the activation of Rap1 necessary to maintain a proper level of N-cadherin at the cell surface (Jossin and Cooper, 2011). After receiving this polarizing cue triggered by Reelin/Rap1/N-cadherin in the MMZ, cells downregulate the signal through degradation of Reelin receptors (Morimura et al., 2005; Uchida et al., 2009), N-cadherin (Kawauchi et al., 2010) and the intracellular adaptor protein Dab1 (Bock et al., 2004; Simó et al., 2010). This downregulation of the signal seems to be important for a correct organization of the cerebral cortex. For example, the ablation of Cullin 5 in migrating neurons results in an accumulation of active Dab1 protein and a cortical layering defect (Feng et al., 2007) while a stabilized mutant Dab1, which resists Cul5-dependent degradation, causes a similar phenotype (Simó et al., 2010).

It is worth noting that rescue experiments showed that activation of the Rap1/N-cadherin pathway only partially rescues the phenotype at the MMZ due to Reelin inhibition and that activation of Akt (which is activated by Reelin in neuronal culture; Beffert et al., 2002; Jossin and Goffinet, 2007) is also needed for a complete rescue (Jossin and Cooper, 2011).

Regulation of cell surface receptors like N-cadherin involves endocytosis, exocytosis, recycling and actin cytoskeleton. As such, molecules known to be involved in those functions such as RalA/B, Rac1 and Cdc42 are implicated in the Rap1-dependent polarization of multipolar neurons (Jossin and Cooper, 2011). Others like Rab5 and Rab11 were also involved in the regulation of N-cadherin in cortical neurons (Kawauchi et al., 2010).

A polarity model for Reelin was unexpected because the position where Reelin is produced does not seem to be important for its function in the embryonic cortex. Forcing the expression of Reelin at the VZ in a Reelin-deficient cortex is able to rescue an early aspect of the reeler phenotype (Magdaleno et al., 2002) and adding Reelin in the culture medium of reeler brain slices is able to rescue the organization of the CP (Jossin et al., 2004). Therefore, and as suggested before (Jossin, 2011), Reelin might rather act as a permissive signal allowing neurons to respond to another polarizing cue that still needs to be discovered.

Ephrin guidance factors and their Eph receptors also regulate the directionality of multipolar migration (Torii et al., 2009; Dimidschstein et al., 2013). Ephrins are cell-surface proteins that trigger a forward signal when binding to Eph family receptors present on other cells. In turn, Eph receptors can also trigger a signal to ephrins through a process of reverse signaling (Arvanitis and Davy, 2008). Eph receptors and ephrins are grouped into class A and class B based on their degree of sequence similarity and binding affinities, with ephrin-A binding to EphA receptors and ephrin-B binding to EphB receptors (Flanagan and Vanderhaeghen, 1998). It was first shown that the Ephrin-A/EphA forward signaling controls the lateral distribution of neurons by promoting a wider tangential migration during the multipolar stage (Torii et al., 2009). Later on it was demonstrated that ephrin-B1 reverse signaling may have an opposite effect and restricts the tangential migration of mutipolar neurons at the MMZ (Dimidschstein et al., 2013; Figure 2). This function however does not seem to be easily explained by pro-adhesive effects of Ephrin/Eph interactions (Dimidschstein et al., 2013). In this article, P-Rex1, a guanine-exchange factor for Rac3 was identified and shown to be required for the control of the lateral migration.

One intriguing question is whether Reelin and ephrins regulate the directionality of multipolar migration in a same pathway or in parallel. Indeed, an inhibition of the Reelin/NCad pathway does not only reduces the radial movement but also increases the lateral displacement of multipolar neurons (Jossin and Cooper, 2011) similarly to an inhibition of ephrin B. Unfortunately the final lateral dispersion was not investigated in this study. A genetic link between the Reelin signaling and ephrin Bs has been suggested (Sentürk et al., 2011). However another team could not confirm the genetic interaction between ephrin B and the canonical Reelin pathway in the radial positioning of cortical neurons (Pohlkamp et al., 2016). Further investigation is needed to definitively answer this question.

According to the concept of radial units, sister cells move from the VZ to the CP where they form functional units arranged as mini-columns (Rakic, 1988; Gao et al., 2013). Multipolar migration and the lateral dispersion that occurs during this critical stage may play an important physiological role since clonally related neurons establish preferential connectivity with each other and can share similar functional properties (Yu et al., 2009, 2012; Li et al., 2012). This seems to be relevant in terms of human health as inappropriate neuronal positioning and abnormal columnar organization have been reported in post-mortem analysis of cortical tissue from subjects with neuropsychiatric disorders and have been related to aging (Buxhoeveden and Casanova, 2002; Casanova and Tillquist, 2008; van Veluw et al., 2012).

### Specification of the Axon at the IZ

In the past, most of the studies investigating axon/dendrite were carried out using dissociated cultures of rodent hippocampal and cortical neurons placed on a two-dimensional artificial substrate (Dotti et al., 1988; Craig and Banker, 1994; Tahirovic and Bradke, 2009). In this system neurons first extend several neurites during what is called stages 1 and 2. Then starts a phase of asymmetric growth (stage 3) during which one of the neurites differentiates into an axon. The main advantage of this system is to be in a well-controlled environment simpler than the *in vivo* situation.

Indeed, very few external signals are present in dissociated cell cultures and the neurons break symmetry randomly. This allowed the identification of several intracellular signaling pathways important for the polarization of the axon/dendrite axe *in vitro* including small GTPases, scaffolding proteins, microtubule-associated proteins, kinases and phosphatases (Arimura and Kaibuchi, 2007; Villarroel-Campos et al., 2016). *In vivo*, however, neurons are generated within a highly oriented three-dimensional tissue. Neuronal polarity is therefore most probably the result of a complex interaction between extracellular cues and intrinsic cell polarity pathways. Extracellular factors such as BDNF and IGF-1 exogenously applied to neuronal cultures have been suggested to initiate the specification of the axonal polarity (Sosa et al., 2006; Shelly et al., 2007). But axon formation was not affected in mice lacking those extracellular cues or their specific receptors (Klein et al., 1993; Jones et al., 1994; Kappeler et al., 2008). On the other hand, TGF-beta signaling has been shown to specify the axon *in vitro* and *in vivo* (Yi et al., 2010).

Several intracellular signaling proteins involved in axon/dendrite polarity *in vitro* have been confirmed *in vivo*: in the double knockout mice for SAD-A and SAD-B kinases, axons were difficult to distinguish from dendrites and dendrites were abnormally oriented. Moreover, this polarity defect did not result from a growth deficiency (Kishi et al., 2005). Lkb1 is an upstream kinase for SAD-A and SAD-B and conditionally deleting Lkb1 resulted in a loss of axon formation (Asada et al., 2007; Barnes et al., 2007; Shelly et al., 2007). Cortical neuron-specific Par3 inhibition using *in utero* electroporation impaired axon/dendrite polarization *in vivo* (Funahashi et al., 2013). Genetic ablation of Cdc42 in the brain strongly suppressed axon formation *in vivo* and in culture, through modulation of actin dynamics (Garvalov et al., 2007). The implication of c-Jun amino-terminal kinase (JNK) enzymes in axon specification and elongation has been described *in vitro* (Oliva et al., 2006; Dajas-Bailador et al., 2008; Eminel et al., 2008; Sun et al., 2013). JNK is a family of three partially redundant isoforms and involved in apoptosis during early brain development (Kuan et al., 1999), rendering difficult the *in vivo* analysis of their contribution in axonal specification and elongation. Nevertheless, JNK1 mutant mice exhibit a defect in the maintenance of axonal integrity (Chang et al., 2003). Overall, these results confirm certain polarity genes as vital for axon/dendrite polarity establishment or maintenance *in vivo*.

At the IZ multipolar neurons migrate through horizontally oriented axons belonging to earlier-born neurons already installed in the CP. It has been suggested that a TAG1-dependent interaction between multipolar neurons and those axons helps the specification of an axon from multipolar cells (Namba et al., 2014). However, another study proposed that the interaction between radial glial fibers and the migrating neuron directs axon formation at the opposite side from the contact site (Xu et al., 2015). It is possible that both types of interactions are necessary.

In mid to late born neurons *in vivo*, axons are specified at the multipolar stage just before neurons resume a bipolar migration (de Anda et al., 2010; Hatanaka and Yamauchi, 2013; Sakakibara et al., 2014; Figure 1). It is therefore tempting to speculate that the specification of the axon is necessary for the polarization of migrating multipolar neurons towards the CP. However several studies suggest that this is not the case. Indeed, even though there is a defect in axonal growth in Rap1 conditional KO mice (Shah et al., 2016), an acute but partial inhibition of Rap1 affects the polarization of multipolar cells towards the CP without an apparent effect on axonal growth (Jossin and Cooper, 2011). Therefore the defect in the orientation of multipolar migration observed is independent of axonal growth. Similarly, Cdk5 is required for the multipolar to bipolar transition but these neurons still can form axons (Ohshima et al., 2007). Along the same line, the multipolar to bipolar transition is not affected by the lack of an axon: in the absence of LKB1 and SAD kinases or of TGF-beta signaling, migrating neurons failed to produce an axon even though they were able to become bipolar and extend a leading process (Barnes et al., 2007; Yi et al., 2010). Therefore it seems that polarization events necessary for: (1) the specification of an axon; (2) the directionality of multipolar neuronal migration; (3) the multipolar to bipolar transition; and (4) the directionality of bipolar neuronal migration all use at least some mechanisms that are distinct *in vivo*.

In humans, several disorders such as autism, schizophrenia, bipolar disorder, mental retardation and some forms of epilepsy are thought to have a perturbed cortical circuitry (Francis et al., 2006). Neuropathological observations in postmortem brain samples include subtle disturbances of cortical lamination and subcortical axonal morphology. Moreover, defects in proteins involved in the specification of axonal-dendrite axe of polarity lead to connectivity-related disorders (Maussion et al., 2008; Corbett et al., 2010; McFadden and Minshew, 2013; Nieto et al., 2013; Quach et al., 2015).

### Centrosome

The position of the centrosome plays an important role in many polarization events. In neuronal culture *in vitro*, its localization specifies the future axon and therefore determines the axon-dendrite polarity (de Anda et al., 2005). *In vivo* the axon of cortical excitatory neurons is specified during the multipolar stage of migration (Shoukimas and Hinds, 1978; Hatanaka and Yamauchi, 2013). Then the axonal growth takes place in parallel with neuronal migration (Schwartz et al., 1991; Hatanaka and Murakami, 2002). A previous study suggested that centrosome and Golgi apparatus position plays an instructive role in specifying the location of axonal outgrowth (de Anda et al., 2010). Then they move away from the growing axon in order to play their role in instructing the orientation of neuronal migration (Shu et al., 2004; Tanaka et al., 2004; Tsai et al., 2007), suggesting that they are not necessary for axonal growth once it is specified *in vivo*. This is in agreement with a study showing that the centrosome is dispensable for axonal extension in hippocampal cell culture (Stiess et al., 2010).

It should be noted that the situation can be different in different models such as in the zebrafish retinal ganglion cells where the centrosome does not predict the site of axon emergence (Zolessi et al., 2006) or in the dsas-4 and dsas-6 mutant flies (with no centrioles) where the development of axons is quite normal (Basto et al., 2006). However, it is possible that these flies still possess the pericentriolar material or that the Golgi apparatus might compensate for the lack of centrioles as a site of microtubules nucleation (Efimov et al., 2007).

The centrosome is also important for orienting the migration of cortical neurons, especially during the locomotion of bipolar neurons along glia within the CP. Here the movement is saltatory (Nadarajah et al., 2001) with repetitions of a two-step cycle: the centrosome moves forward in the leading process then the nucleus follows (Tsai et al., 2007). The centrosome seems to regulate nuclear translocation. Microtubules surround the nucleus in a fork-like shape and extend to the centrosome (Xie et al., 2003) while SUN and Nesprin proteins connect the centrosome to the nucleus via microtubules during radial bipolar migration (Zhang et al., 2009). Lis1 and dynein, a microtubule minus-end-directed motor, are responsible for coupling nuclear translocation and centrosome movement (Tsai et al., 2007). Microtubules are oriented with their minus-end towards the centrosome and dynein is concentrated at a dilation in the leading process and near the nucleus. Therefore, Dynein may pull the centrosome forward toward the dilation and the nucleus toward the centrosome (Tsai et al., 2007). In humans, abnormalities in centrosomal proteins such as lis1 cause lissencephalies and subcortical band heterotopia (Cardoso et al., 2000; Sicca et al., 2003).

### Signals for Multipolar to Bipolar Transition

After making their way through the MMZ, multipolar neurons transform into bipolar neurons. They invade the RMZ and are polarized towards the pial surface. They exhibit a thick leading process and a thin axon growing at the rear (Noctor et al., 2001; Hatanaka and Murakami, 2002). This type of migration depends on radial glia fibers as substrate. An important step here is the transition from multipolar to bipolar migration. Many changes occur: the substrate for migration (from glial fiber-independent to glial fiber-dependent), the shape of the cell (multipolar to bipolar) and the speed of migration (from about 4 microns per hour to around 12 microns per hour when investigated in organotypic brain slice cultures). Several proteins including kinases, small GTPases, regulators of the cytoskeleton or membrane proteins have been discovered to be involved in this transition (Kawauchi et al., 2003; Nagano et al., 2004; LoTurco and Bai, 2006; Ohshima et al., 2007; Young-Pearse et al., 2007; Sapir et al., 2008; Sun et al., 2010; Nakamuta et al., 2011; Pacary et al., 2011; Westerlund et al., 2011; Lee et al., 2012; Falace et al., 2014; Inoue et al., 2014; Jacobshagen et al., 2014). Because of the diversity of molecules implicated, multiple mechanisms might explain the defect in the transition observed when they are inhibited. However it appears that a common feature is a direct or indirect regulation of the cytoskeleton. We will not discuss them here and would like to refer the readers to other recent reviews on the subject (Cooper, 2014; Ohtaka-Maruyama and Okado, 2015).

We have seen earlier that molecular pathways are involved in polarizing the movement of multipolar neurons towards the CP. Once they reach the top of the MMZ, they initiate their attachment to the radial glia fibers which might give them the signal to transform into bipolar cells but there is no hard proof for this and cells might become bipolar before they attach to the fibers. Since multipolar neurons switch direction of migration a few times while their net movement is oriented towards the CP, it is also possible that signals that trigger the multipolar to bipolar transition are actually reinforcing the polarity of the movement towards the CP already present in the multipolar cells.

### Bipolar Migration

During bipolar migration, neurons exhibit a constant polarized migration towards the pial surface and migrate along radial glia fibers in order to reach the top of the CP (Noctor et al., 2001; Hatanaka and Murakami, 2002). Because the substrate of migration is the radial glia, it has been assumed that directionality was conferred by the fibers. It was shown that the interaction with radial fibers is mediated by Connexin 43 (Elias et al., 2007). Connexin 43 is expressed at the contact points between radial glia and migrating neurons. It interacts with the cytoskeleton enabling the stabilization of the leading process along the fibers. Interfering with Connexin 43 expression inhibits neuron migration into the RMZ (Elias et al., 2007).

Adhesion to the radial glia fibers is not the only factor regulating bipolar migration. Extracellular signals also come into play. At least one secreted cue is important to keep the directionality of the bipolar migration. Semaphorin-3A is a secreted factor expressed by the superficial layers of the CP that diffuses in a descending gradient across the cerebral wall. Semaphorin-3A receptors neuropilin-1 and several Plexins are expressed by migrating neurons (Polleux et al., 2000; Chen et al., 2008). Inhibition experiments and time lapse video microscopy showed that Sema3a and its receptors are important to keep bipolar neurons polarized towards the CP (Chen et al., 2008). Inhibited neurons showed a mis-oriented bipolar migration and exhibited a leading process pointing to the wrong direction. They accumulated within the different layers of the CP and at the border between the MMZ and RMZ. Modification of the location of Sema3A gradient in slice culture assays also affected the polarity of migration suggesting that it is acting as an attractive molecule rather than affecting the interaction with the radial fibers (Chen et al., 2008).

Other extracellular signaling proteins are important for a correct bipolar migration but might not do so by regulating the directionality of migration. Tgf-beta extracellular signaling is required for radial migration. Neurons inhibited for the receptor TgfbR2 are able to proceed through the multipolar to bipolar transition, and exhibit a correctly oriented leading process but are unable to migrate and remain stuck in the lower part of the CP (Yi et al., 2010).

As explained in “Extracellular Signals for the Directionality of Multipolar Migration” Section, once Reelin/NCad signaling has triggered polarization of multipolar neurons towards the CP, it is no longer required for the radial glia-dependent bipolar migration (Jossin and Cooper, 2011). But as soon as bipolar migrating neurons reach the top of the CP, they must proceed through a glia-independent terminal translocation during which neurons detach from the radial glia fiber and the leading process shortens as the soma moves upward (Nadarajah et al., 2001; Sekine et al., 2011). This time Reelin and Rap1 rather regulate integrin receptors while N-cadherin is not involved (Sekine et al., 2012). Reelin signaling in neurons reaching the top of the CP promotes neuronal adhesion to fibronectin through integrin α5β1. The activated integrin then allows final somal translocation (Sekine et al., 2012). Of note, earlier-born neurons also undergo glia-independent somal translocation but this does involved Reelin/Rap1/NCad (Gil-Sanz et al., 2013). For these early-born cells, somal translocation depends on the interaction between the leading process of migrating neurons and Cajal-Retzius cells. However, inhibition of this interaction did not affect cell polarization (localization of the Golgi apparatus ahead of the nucleus) and leading process extension (Gil-Sanz et al., 2013). The reeler phenotype in the cortex might therefore be a combination of the absence of Reelin’s multiple functions at different stages of radial migration.

Finally, inhibition of the intracellular protein JNK inhibits bipolar migration but polarity of migration does not seem to be affected. JNK inhibition does not affect the multipolar to bipolar transition. Neurons with inhibited JNK exhibit a leading process oriented in the correct direction but it is twisted and unable to lead the migration resulting in cells stalled at the bottom of the RMZ (Kawauchi et al., 2003).

## Concluding Remark

Glutamatergic excitatory cortical neurons are born near the ventricle then travel long distances in order to reach their final destination within the cortex. Polarization events play critical roles during the embryonic development of the cortex. First, molecular and cellular polarity is essential for NSCs to self-renew and produce the adequate number of neurons at a correct timing. Then a precise coordination of extracellular and intrinsic polarity signals is necessary for the definition of the directionality of migration. Finally the axon/dendrite polarization occurs during the multipolar migration.

Advances in our understanding in polarization events have been made by using techniques to manipulate signaling pathways such as *in utero* electroporation and conditional gene knockout and by using live imaging techniques. However, an important challenge still facing the field is to discover how all these extracellular cues and intrinsic signals precisely regulate polarization events in a coordinated manner *in vivo*.

## Author Contributions

YJ wrote the manuscript. EK and AC substantially contributed to the manuscript through discussions.

## Conflict of Interest Statement

The authors declare that the research was conducted in the absence of any commercial or financial relationships that could be construed as a potential conflict of interest.
